# C-reactive protein upregulates the whole blood expression of *CD59* - an integrative analysis

**DOI:** 10.1371/journal.pcbi.1005766

**Published:** 2017-09-18

**Authors:** Kaido Lepik, Tarmo Annilo, Viktorija Kukuškina, eQTLGen Consortium, Kai Kisand, Zoltán Kutalik, Pärt Peterson, Hedi Peterson

**Affiliations:** 1 Institute of Computer Science, University of Tartu, Tartu, Estonia; 2 Institute of Social and Preventive Medicine, Lausanne University Hospital, Lausanne, Switzerland; 3 Swiss Institute of Bioinformatics, Lausanne, Switzerland; 4 Estonian Genome Center, University of Tartu, Tartu, Estonia; 5 Molecular Pathology, Institute of Biomedical and Translational Medicine, University of Tartu, Tartu, Estonia; 6 Quretec Ltd, Tartu, Estonia; University of Chicago, UNITED STATES

## Abstract

Elevated C-reactive protein (CRP) concentrations in the blood are associated with acute and chronic infections and inflammation. Nevertheless, the functional role of increased CRP in multiple bacterial and viral infections as well as in chronic inflammatory diseases remains unclear. Here, we studied the relationship between CRP and gene expression levels in the blood in 491 individuals from the Estonian Biobank cohort, to elucidate the role of CRP in these inflammatory mechanisms. As a result, we identified a set of 1,614 genes associated with changes in CRP levels with a high proportion of interferon-stimulated genes. Further, we performed likelihood-based causality model selection and Mendelian randomization analysis to discover causal links between CRP and the expression of CRP-associated genes. Strikingly, our computational analysis and cell culture stimulation assays revealed increased CRP levels to drive the expression of complement regulatory protein *CD59*, suggesting CRP to have a critical role in protecting blood cells from the adverse effects of the immune defence system. Our results show the benefit of integrative analysis approaches in hypothesis-free uncovering of causal relationships between traits.

## Author summary

Chronic inflammation is associated with chronic diseases, morbidity and mortality while lower base inflammation levels are thought to be predictive of healthy aging. Thus, to pursue a long and healthy lifespan, it is essential to understand the inflammatory regulatory mechanisms. To that end, we studied the functional role of C-reactive protein (CRP)–an inflammatory biomarker that is used to measure cardiovascular risk in clinical practice. There is evidence for a strong genetic component of elevated CRP levels but it is still unclear if it has a direct impact on the processes that lead to inflammatory diseases. In order to elucidate the function of CRP in the blood, we used statistical methods for causal inference to infer causal relationships between changes in CRP and gene expression levels. Our statistical analysis and cell culture experiments suggest that CRP drives the expression of complement regulatory protein *CD59*. Thus, CRP can have a functional role in protecting human blood cells from the adverse effects of the immune defence system.

## Introduction

Increased levels of C-reactive protein (CRP) in the blood are associated with tissue injury, infections and inflammation [1]. In addition to acute bacterial and viral infections, chronically elevated CRP levels are predictive of multiple diseases associated with inflammatory processes, e.g. cardiovascular disease (CVD). Therefore, CRP is used as a biomarker to diagnose CVD and other inflammatory diseases [2–4]. Furthermore, a recent large-scale Mendelian randomization (MR) study has shown a possible causal relationship between CRP and several complex traits, most notably a protective effect against schizophrenia [5]. However, little is known about the mechanisms of the underlying inflammatory processes and the interactions between different risk factors that either prevent or lead to a disease.

In the past years, genome-wide association studies (GWAS) have identified thousands of disease-associated genetic loci, and a GWAS meta-analysis of CRP levels in over 80,000 individuals found a number of allelic variants in genes implicated in pathways related to metabolism and immune system [6]. Altogether, these studies have demonstrated a strong genetic component in chronic inflammatory processes. However, the identification of genetic variants without knowing their functional relevance has not been sufficient to tackle disease-informed genetics and provide intervention measures against complex diseases. Hence it is necessary to integrate different omics data and move beyond associations. Causal inference methods and multi-omics approaches have already been applied successfully in the analysis of complex traits, e.g. obesity, cancer and coronary artery disease [7–10].

Recent methodological approaches in causal inference include finding the best-fitting model from the set of previously defined possible causal models using maximum likelihood [10–14], testing for partial correlation criteria based on the theory of d-separation [15, 16], both of these techniques together [17, 18], and MR [19–22]. Especially MR has been increasingly popular of late but requires thousands of samples to achieve adequate statistical power even at nominal significance level 0.05 [22] and is therefore not feasible for hypothesis-free testing in smaller samples. Model selection-based methods do not necessarily rely on p-values and can have more power but are prone to false positive findings [15, 23]. To overcome this trade-off, we propose a combined approach where we first identify a list of candidate causal relationships using a model selection-based approach and then apply MR on this candidate list to disentangle true positive findings.

**Fig 1:**
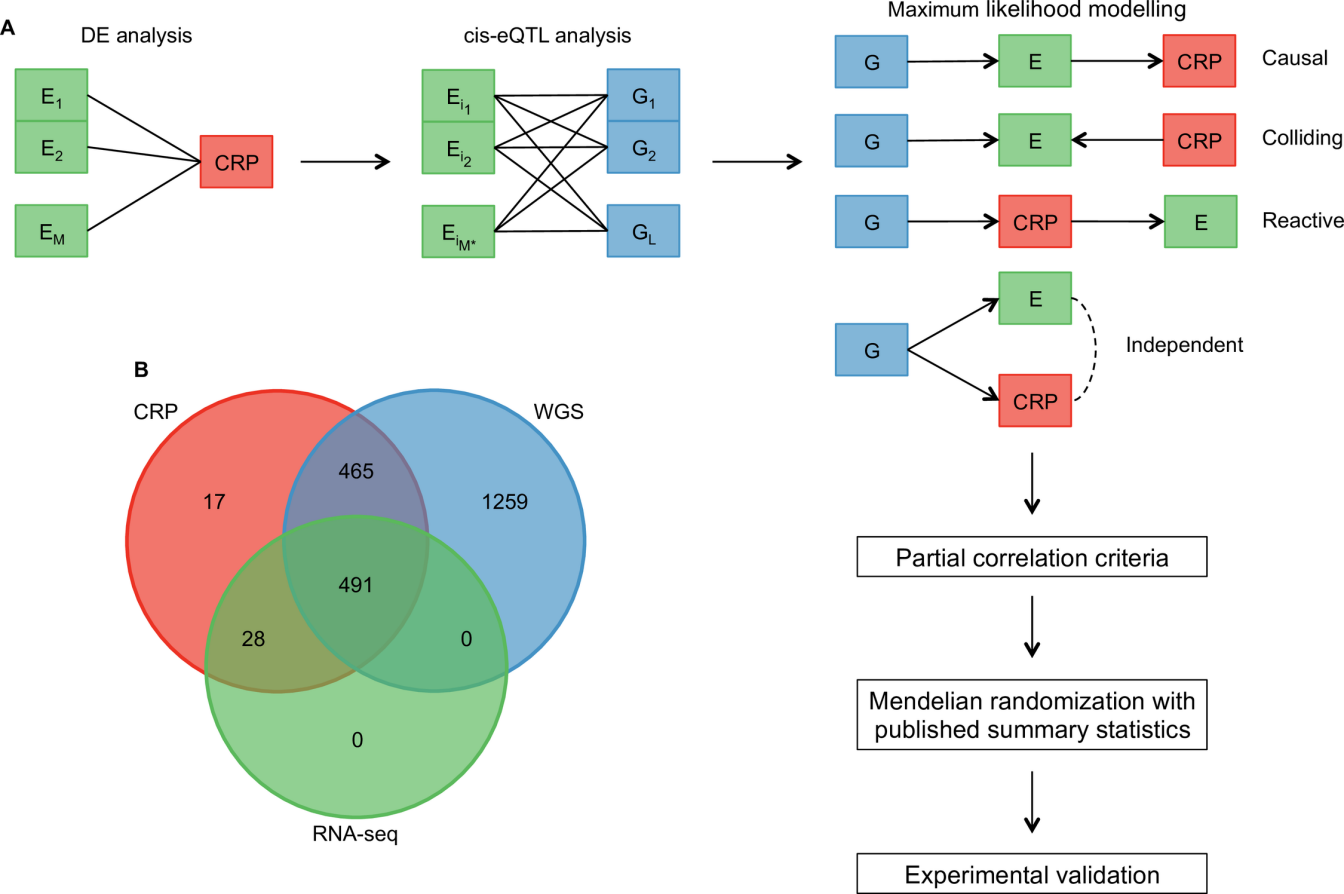
Pairwise modelling pipeline of whole genome sequencing (WGS), RNA sequencing (RNA-seq) and C-reactive protein (CRP) data. (A) First, we identified genes whose expression levels (denoted by E) were significantly associated with CRP. Second, we used these genes to perform a *cis*-eQTL analysis and extract SNPs (denoted by G) that act on the expression of those genes. Third, for each triplet (G, E, CRP), we used maximum likelihood to select the best supported model out of a limited number of possible models–given that G is correlated with E, E is correlated with CRP and assuming directed acyclic graphs. The dashed edge in model IV indicates that either E acts on CRP or vice versa–these two models are Markov equivalent so we cannot differentiate between them. Fourth, we ensured that the best candidate models fulfilled necessary partial correlation criteria. Fifth, we subjected the best candidates to MR analysis where the instruments were chosen from published GWAS summary statistics. Finally, we validated the findings using cell culture stimulation assays. (B) Venn diagram of available sample sizes.

Here we combined data on genotype, transcriptome and CRP levels to get further insight into the molecular mechanisms regulating CRP concentration. We hypothesized that understanding the complex genetic architecture of the molecular functions behind CRP levels can be aided by overlapping the genetic basis of CRP and the genetic basis of gene expression variability. To this end, we have performed a multi-step analysis procedure. We identified and described the set of genes whose expression levels are associated with CRP levels and then used genotype data to determine the potential causal structure between the expression of these genes and CRP by maximum likelihood. We ensured that the proposed models satisfied all the necessary partial correlation criteria, and then used MR and independent data to decide on true causal findings (Fig 1A). We identified a causal effect of CRP concentration on *CD59* expression in whole blood which we validated experimentally. The main study was conducted on 491 individuals from the Estonian Biobank cohort [24] whose genomes and transcriptomes in whole blood have recently been profiled using whole genome sequencing (WGS) [25] and RNA sequencing (RNA-seq) techniques (Fig 1B).

## Results

### CRP-associated genes are implicated in pathways related to immune system

To find CRP-associated genes, we performed a differential expression analysis using the limma-voom framework [26]. However, instead of dividing continuous CRP values into two or more bins, we simply used the close-to-normally distributed log-transformed CRP values as a continuous predictor. Binning a continuous variable would result in reduced power to detect true associations [27]. The models were adjusted for age, sex, body mass index (BMI), blood composition and principal components (PCs) both from the genotype and gene expression data to account for population structure and hidden batch effects.

Controlling the false discovery rate (FDR) at 0.05, we identified 1,614 genes whose expression values were significantly associated with CRP concentrations in the blood (S1 Table). Of all the CRP-associated genes, 1,108 were positively and 506 negatively correlated with CRP. As expected, we observed a high proportion of interferon-stimulated genes, 738 in total (45.7%), which are known to be induced by infections and inflammatory processes [28] (S1 Fig). By a considerable margin, the most significant CRP-associated gene was *FAM20A* (adjusted p = 7.5×10^−17^), followed by *UPP1*, *FCGR1A*, *LDHA* and *MTHFD2* (Table 1). Similarly to the *CRP* gene, *FAM20A* is most highly expressed in the liver. *FAM20A* is known to be involved in biomineralisation of teeth and mutations in this gene have previously been linked to dental defects and enamel renal syndrome [29, 30]. Pathway enrichment analysis with g:Profiler [31] showed that CRP-associated genes are overrepresented in immune system processes, particularly in innate immune system and interferon signalling pathways, as well as in NOD-like receptor signalling pathway (S2 Table).

**Table 1:** Top 10 CRP-associated genes. CRP-gene expression association effect sizes (Beta) with 95% confidence intervals (CI) and p-values adjusted for 5% FDR (Adjusted p-value) are shown.

Gene	Chromosome	Beta	95% CI	Adjusted p-value
*FAM20A*	17	0.51	0.40–0.61	7.5×10^−17^
*UPP1*	7	0.10	0.07–0.12	2.6×10^−11^
*FCGR1A*	1	0.28	0.21–0.36	5.6×10^−10^
*LDHA*	11	0.06	0.04–0.08	5.6×10^−10^
*MTHFD2*	2	0.08	0.06–0.10	8.7×10^−10^
*MS4A4A*	11	0.19	0.14–0.24	1.3×10^−9^
*DUSP3*	17	0.08	0.06–0.10	1.3×10^−9^
*GYG1*	3	0.12	0.09–0.15	1.3×10^−9^
*FBXO6*	1	0.14	0.10–0.18	4.0×10^−9^
*IFI27*	14	0.58	0.42–0.74	4.2×10^−9^

### *Cis*-eQTL analysis of CRP-associated genes

To study the genetics influencing the expression of the CRP-associated genes, we performed an expression quantitative trait locus (eQTL) analysis. We used the same set of covariates as before with the addition of one dummy variable coding for different batches of WGS data. We limited our search to single nucleotide polymorphisms (SNPs) located within 250 kb of the genes. For each pair of SNP and gene expression values, we tested whether an additional minor allele of the SNP has a significant additive effect to the level of gene expression. In total, we performed 1,821,299 tests. We identified 39,507 eQTLs for 470 different genes (S3 Table). To validate our findings, we compared the results against the *cis*-eQTLs reported in whole blood by the GTEx Consortium (version V6p) [32]. We could replicate at least one eQTL for 313 out of the 470 genes (66.6%), altogether 20,536 SNP-gene pairs. This shows good concordance, despite several differences in the study designs and the relatively low power of both studies. Compared to the eQTLs reported by Westra *et al*. [33], we replicated at least one eQTL in 273 genes (58.1%), altogether 8,998 SNP-gene pairs. The considerably smaller replication rate here is likely to be due to the differences between array- and sequencing-based expression profiling (e.g. lowly expressed genes are likely not replicable in microarray-based eQTL studies) [34].

### Causal relevance between CRP and gene expression by maximum likelihood

In the previous steps, we established genes that are associated with CRP through their expression values and we also identified eQTLs for these genes, creating a set of SNP, gene expression and CRP triplets. Assuming directed acyclic graphs, this leaves only a limited number of possible models that these triplets can be functionally acting by (Fig 1A). To determine the most likely causal structure underlying these triplets, we performed likelihood-based causality model selection [10]. That is, we modelled the joint distribution of all possible triplet models by maximum likelihood and determined which was best supported by our data in terms of minimal values of the Akaike information criterion (AIC). To eliminate the situations where both CRP and gene expression were driven by known confounding factors, we performed the analysis on covariate-adjusted CRP and expression values, using the same set of covariates as before (except for gene expression PCs in the case of CRP). As many of the eQTL SNPs were in high linkage disequilibrium (LD) with each other, we first identified independent eQTLs for each gene using stepwise multiple regression, starting from the strongest *cis*-eQTL. This is a standard approach for discovering independent loci [35]. In total, we found 536 independent eQTLs for 470 different genes.

**Fig 2:**
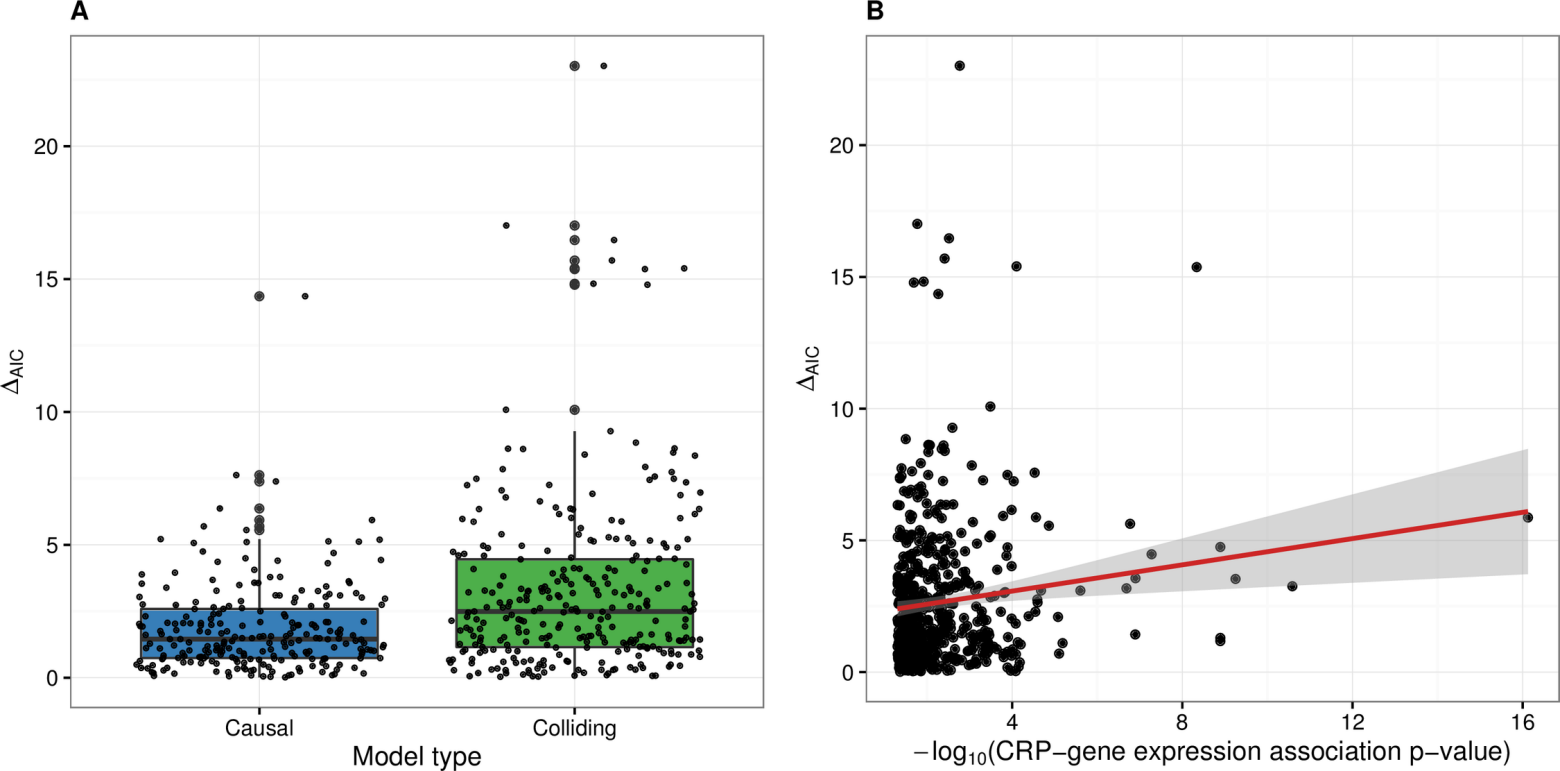
Analysis of Δ_AIC_ values. (A) Differences in the Akaike information criterion values between the causal and colliding models (Δ_AIC_) in triplets best supported by either model. On average, the colliding models have higher Δ_AIC_ values. This indicates that we are more likely to identify genes whose expression is in some way regulated by CRP. (B) Scatter plot of Δ_AIC_ values against the CRP-expression association p-values with a linear trend and 95% confidence interval. Despite a small positive trend, we can observe that higher correlation does not necessarily translate to more evidence of a causal effect.

For 283 out of 536 triplets tested, the difference in AIC values between the causal and colliding models (Δ_AIC_) was less than 2, which does not give enough evidence to support one model over the other [36]. Among the remaining 253 triplets, 81 showed stronger evidence for the causal model, 163 for the colliding model and 9 for the independent model. Unsurprisingly, the reactive model never achieved the smallest AIC, due to the selection bias of the SNPs. Altogether, 223 unique genes were represented in the 253 triplets. There were 21 genes with multiple independent eQTLs and 15 of them were supported by a single model, showing good consistency (S4 Table). On average, triplets supported by the colliding model showed higher Δ_AIC_ values (Fig 2A). This indicates that the colliding models are of higher quality in our analysis. Genes best supported by these colliding models were enriched in Gene Ontology terms for response to external stimulus and stress (S5 Table).

We could also observe that more significant association p-values between CRP and gene expression do not necessarily translate to greater Δ_AIC_ values (Fig 2B). This result reinforces that many of the correlations resulting from ordinary differential expression analysis are likely to rise due to unmeasured common confounding and care should be taken when interpreting such results.

### CRP upregulates *CD59* expression in blood

To be able to clearly isolate genes whose expression with respect to CRP conforms to either the causal or colliding model (i.e. whether gene expression drives CRP or vice versa), we would expect a clear difference in the AIC values of corresponding triplet models, so we considered only triplets with Δ_AIC_ ≥ 10 as candidates. This Δ_AIC_ threshold corresponds to probability 1 –e^-5^ > 0.99 that the model with the smaller AIC is more likely [36]. We further required that the models suggested by the maximum likelihood procedure satisfied partial correlation criteria (S4 Table). More specifically, for the causal models we expect to observe at least nominally significant association between the SNP and CRP values, but not if we conditioned on gene expression values. On the other hand, for the colliding models we expect to observe an association between SNP and CRP conditional on gene expression, but not otherwise.

The Δ_AIC_ and partial correlation criteria already provide evidence of causality but unmeasured common confounding can be an issue and lead to overconfident claims. Therefore, we subjected all the best models to MR analysis using published CRP summary statistics [6]. For causal models, we checked whether the eQTL was significantly associated with CRP in the published data. For colliding models, we selected 16 out of 18 CRP-associated SNPs from the CRP meta-analysis [6] (the remaining 2 SNPs had a minor allele frequency of 2.2% and no individual in our sample had two minor alleles of these SNPs) and performed association tests between these SNPs and gene expression in the Estonian data, looking for enrichment of small p-values. To increase power, we also combined the 16 SNPs into a genetic risk score (GRS_CRP_) using published effect sizes as weights. To estimate the causal effect, we used the two stage least squares (TSLS) method which is standard in MR analysis [37].

Only ten triplets (1 causal, 9 collider) had Δ_AIC_ at least 10 (Tables 2 and 3). Out of those, *FADS2* was the only gene best supported by the causal model. The corresponding SNP (*rs61897793*) was not present in the CRP meta-analysis so we performed summary statistic imputation [38] (with UK10K as reference panel) to infer the CRP-association statistic. It did not reach nominal significance (p = 0.0996). However, SNPs in the *FADS2* gene have been associated with circulating phospholipid trans fatty acid and plasma phospholipid n-3 fatty acid levels by the CHARGE Consortium [39–41]. CRP has been shown to bind phospholipids through phosphorylcholine [42] and plasma CRP values have been reported to drop with phospholipid-induced agglutination [43]. *FADS2* has a known function in the synthesis of arachidonic acid that is relevant in inflammatory processes and has been associated with both CRP and risk of CVD [44]. Furthermore, SNPs in *FADS2* have also been associated with low-density lipoprotein (LDL) and total cholesterol in European populations [45], in addition to weight and BMI in Greenlanders [46]. LDL-cholesterol and BMI have in turn been causally implicated with risk of CVD [47] and CRP [19]. These results are consistent with a mediated causal indirect effect of *FADS2* on CRP, even though a recent summary-level MR analysis did not identify *FADS2* expression causal to BMI [22]. We could not fully confirm a causal link between *FADS2* expression and CRP in this study, but together with evidence from other studies, our results could warrant further analysis with a larger sample size.

**Table 2:** Gene supported by the causal model with Δ_AIC_ ≥ 10 using the strongest *cis*-eQTL.

Chr	Gene	SNP	A1^i^	N_SNP_	Δ_AIC_	Z-score^ii^	P-value^iii^
11	*FADS2*	*rs61897793*	A	491	14.4	1.647	0.0996

^i^ The effect allele.

^ii^ Imputed Z-score of SNP-CRP association based on the CRP meta-analysis [6].

^iii^ P-value from the CRP summary statistic imputation.

**Table 3:** Genes supported by the colliding model with Δ_AIC_ ≥ 10 for at least one of the independent *cis*-eQTLs.

Chr	Gene	SNP	A1^i^	N_SNP_	Δ_AIC_	Beta (SE)^ii^	P-value^iii^
12	*C3AR1*	*rs2072448*^a^	A	491	23.0	0.19 (0.10)	0.06
9	*HIATL1*	*rs10993177*^b^	G	491	17.0	0.09 (0.05)	0.09
		*rs7863391*^c^	C	491	2.6		
8	*NRG1*	*rs2466077*	T	491	16.5	-0.04 (0.20)	0.83
1	*SEMA4A*	*rs7695*	C	491	15.7	-0.01 (0.05)	0.83
9	*PLGRKT*	*rs2104175*	C	491	15.4	0.09 (0.05)	0.06
**11**	***CD59***	***rs2272064***	**A**	**491**	**15.4**	**0.20 (0.06)**	**0.0012**
19	*FCGBP*	*rs4802064*	T	491	14.8	-0.19 (0.14)	0.17
		*rs4803308*^*d*^	T	491	3.8		
11	*IFITM3*	*rs7942247*	A	490	14.8	0.29 (0.20)	0.15
22	*KREMEN1*	*rs134615*	C	491	10.1	0.06 (0.13)	0.63

^i^ The effect allele.

^ii^ Causal effect estimate and standard error from the MR analysis using CRP-associated SNPs [6] as instruments.

^iii^ P-value from the MR analysis using CRP-associated SNPs [6] as instruments.

^a^ SNP *rs2072449* has equivalent values in our data.

^b^ SNPs *rs138924760*, *rs141639969*, *rs147817734* and *rs56062008* have equivalent values in our data.

^c^ Weaker eQTL of *HIATL1*, independent from *rs10993177* in our data.

^d^ Strongest eQTL of *FCGBP*, independent from *rs4802064* in our data.

**Fig 3:**
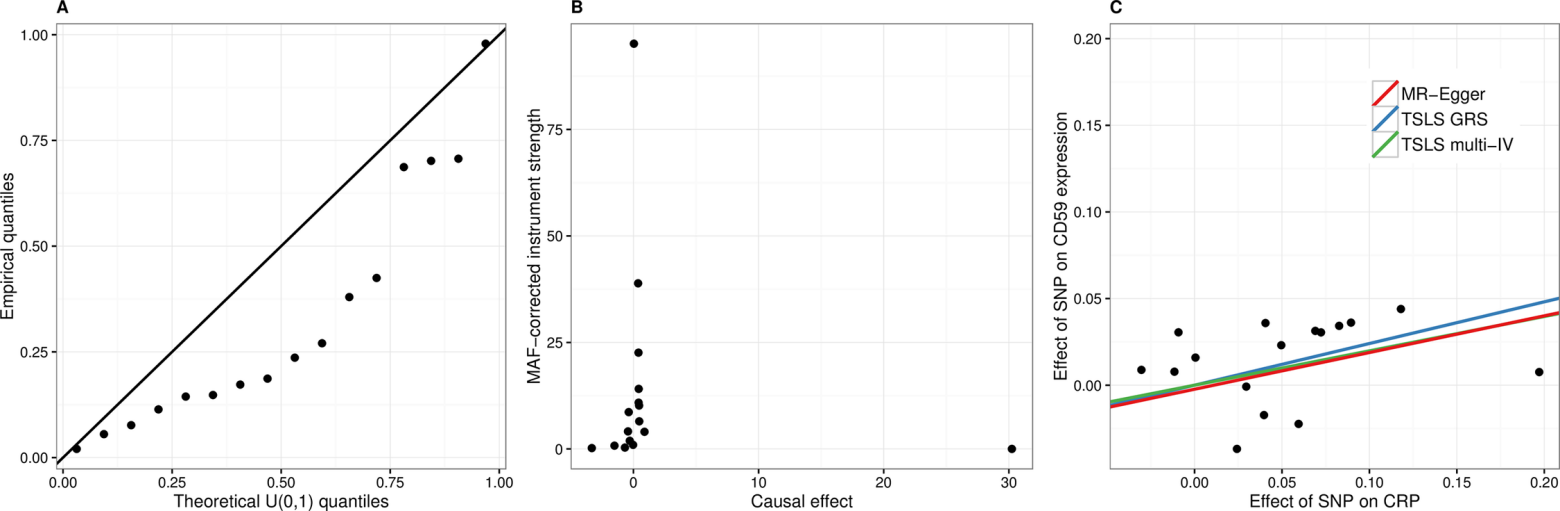
Analytical validation of the causal CRP and *CD59* link. (A) QQ-plot of p-values from the association analysis between CRP-associated SNPs and *CD59* expression. The empirical quantiles are not in line with the theoretical quantiles of the uniform distribution (Kolmogorov-Smirnov p = 0.026) and there is some enrichment of small p-values. (B) Funnel plot of minor allele frequency corrected genetic effects on CRP against causal effect estimates between CRP and *CD59* expression for each CRP-associated SNP. (C) Scatter plot of the genetic effect on *CD59* expression against the genetic effect on CRP. Causal effect slope estimates from the TSLS solutions with the GRS_CRP_ instrument and with all the 16 CRP-associated SNPs as instruments (both forced through zero) are coloured in blue and green, respectively. The bias-corrected slope from the MR-Egger regression is shown in red.

The top genes following the colliding model were *C3AR1*, *HIATL1*, *NRG1*, *SEMA4A*, *PLGRKT*, *CD59*, *FCGBP*, *IFITM3* and *KREMEN1*. Out of these, *CD59* was the only gene that showed enrichment of low association p-values with the 16 individual CRP-related SNPs from the CRP meta-analysis (Fig 3A, Kolmogorov-Smirnov test for uniform distribution p = 0.026). The estimate of causal effect from CRP to *CD59* expression using all the individual SNPs as instruments (beta = 0.20, SE = 0.06, p = 0.0012) was similar to using only GRS_CRP_ as a single strong instrument (beta = 0.24, SE = 0.08, p = 0.0022), showing a similar positive slope in both cases. None of the SNPs nor GRS_CRP_ were correlated with *CD59* expression conditional on CRP values, satisfying the conditional independence assumption of MR. To detect and correct for possible bias from pleiotropy, we calculated the causal effect estimate using each SNP as a single instrument, visualized the individual causal estimates by a funnel plot and performed an MR-Egger test proposed by Bowden *et al*. [21] (Fig 3B and 3C). There was very little directional pleiotropy present (the intercept coefficient from the MR-Egger test was -0.002) and the MR-Egger-corrected causal effect estimate was similar to the TSLS estimates (beta = 0.21). For the other genes, there is less evidence for a causal effect from CRP to expression and instead, unmeasured confounding might be responsible for the elevated Δ_AIC_ values.

**Fig 4:**
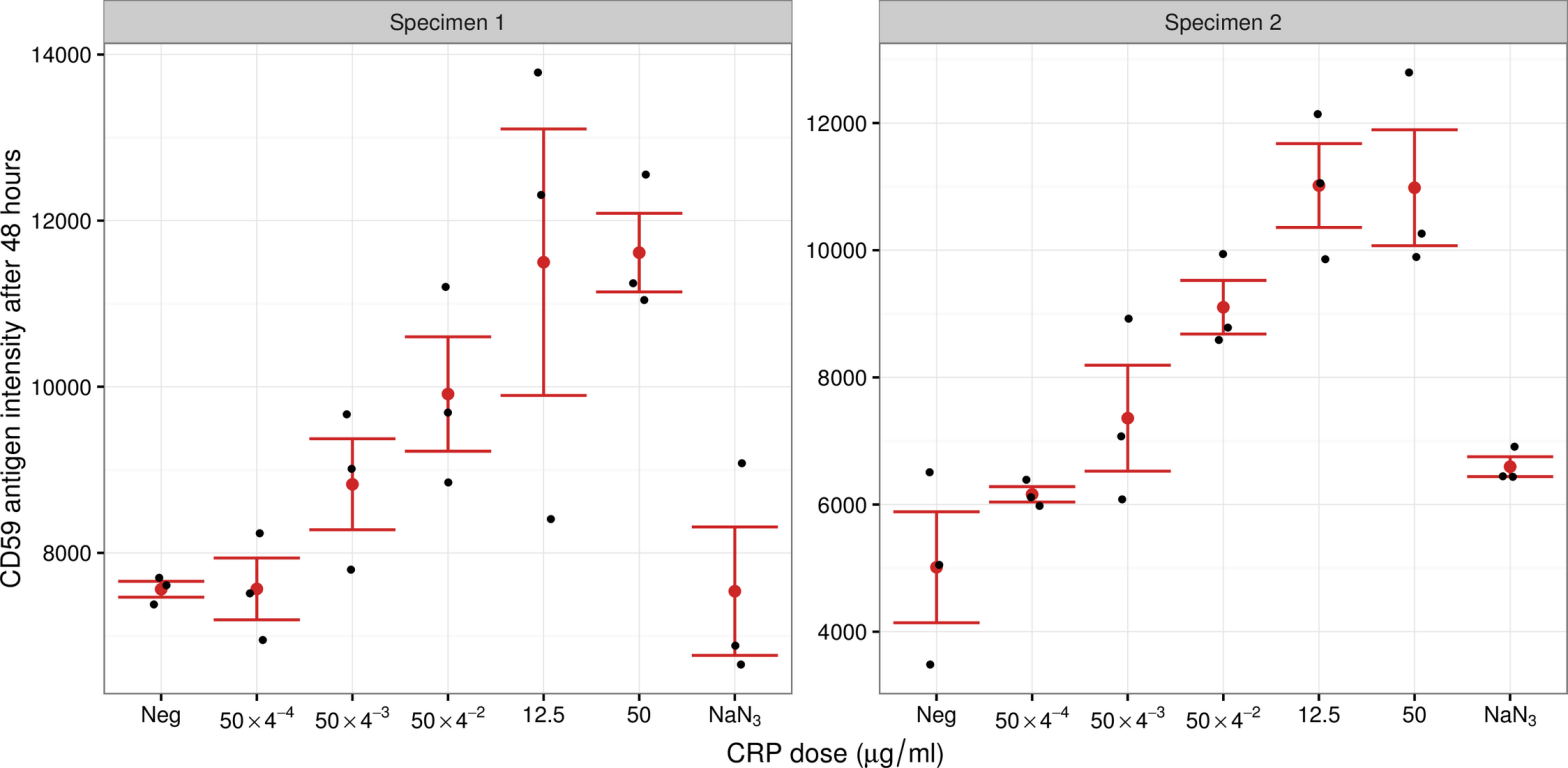
Upregulation of *CD59* surface expression by CRP in cell culture experiments. Peripheral blood cells from two donors were treated with five increasing doses of CRP protein. For negative controls, the cells were not treated with CRP or were treated with additive NaN_3_ only. The *CD59* antigen values were measured after 48 hours and are shown in mean fluorescent intensity units as the arbitrary values of flow cytometry. Black dots represent individual measurements in different replicates, red dots are the averages and whiskers represent ±1 standard errors.

Functionally, *CD59* regulates the complement membrane attack complex (MAC) [48] and has been reported to have a protective effect against atherosclerosis by restricting MAC formation [49]. CRP has also been shown to upregulate *CD59* in endothelial cells [50] and although some of the findings of this paper were later questioned by the effect of a common additive sodium azide (NaN_3_), the upregulation of *CD59* by CRP was not disproved [51]. To further confirm that the expression of *CD59* is upregulated in the blood by elevated CRP levels, we performed cell culture experiments where we stimulated peripheral blood leukocytes with increasing concentrations of CRP (Fig 4). We found a dose-dependent upregulation of *CD59* on cell surface by flow cytometry after 48 hours, which importantly was not present when only NaN_3_ was added to the cell cultures. The dose effect was most prominent in lower doses while reaching a plateau at the concentration of 12.5 μg/ml. A similar trend in increased *CD59* surface levels, albeit slightly lower, was also present after 24 hours (S2 Fig). Altogether, our results indicate a causal role of CRP on *CD59* expression levels.

## Discussion

We identified altogether 1,614 genes that are associated with CRP by their expression values in the blood. Using pathway analysis, we have shown that these genes are enriched in immune system related functions and thus are good candidates to be directly relevant in biological processes concerning CRP. In agreement with their function in innate immune responses, ca 46% of CRP-associated genes comprised interferon-stimulated genes, which have a wide range of activities ranging from control of bacterial and viral infections, upregulation of chemokines and chemokine receptors and regulating blood cellular homeostasis [52]. However, our results suggest that the most significant CRP-associated genes should not be readily interpreted as the most important in terms of causal effects. To find causal relationships, we integrated gene expression and CRP data with genotype data and used a combined analysis approach. First, we applied integrative genomics techniques to filter out a list of candidate causal relationships and then applied Mendelian randomization to determine the final outcome. We report the expression of *CD59* as being causally affected by CRP concentration in the blood, and provide experimental validation of the result.

Our finding of CRP-mediated induction of *CD59* suggests a negative feedback mechanism to protect blood cells against potentially damaging complement responses that are upregulated during infections and inflammation. Ubiquitously expressed *CD59* is a specific inhibitor of complement membrane attack complex (MAC) formation, which is the main effector of complement-mediated tissue damage and leads to osmotic lysis of targeted cells [48, 53]. Through its inhibitory binding to complement members, *CD59* blocks MAC formation and MAC-induced cell lysis. For example, individuals having mutations in *CD59* have decreased capacity to inhibit the complement MAC formation and develop an early-onset hemolytic phenotype associated with vascular disease [54]. Thus, our result provides a new insight into the molecular mechanism of CRP function in protecting human blood cells from the adverse effects of the innate immune defence system, albeit the exact interplay between CRP and *CD59* needs to be determined in further experiments.

We also found that the expression of *FADS2* can be potentially relevant in terms of CRP regulation. There are many known associations between *FADS2* genotypes, lipid levels, inflammatory markers and CVD that together are consistent with a mediated causal effect of *FADS2* expression on CRP. We did not find conclusive evidence of this causal relationship in this study but suggest further analysis to ascertain the interplay between these traits in terms of inflammation and disease.

Our study has several limitations. By selecting candidate triplets using a stepwise analysis approach, we make an implicit assumption that variation in DNA leads to variation in the phenotype in a linear manner. However, it is reasonable to believe that variation in the phenotype values is determined by the combined variation of many factors in multiple omics layers [55]. Further, the triplet models that we considered (including the assumptions) are likely to be simplistic representations of actual relationships between variables and although we accounted for several known covariates and captured technical variation in the data, it is possible that unmeasured variables are acting as confounders in some cases. These drawbacks can yield false positive findings. However, Δ_AIC_ of at least 10 provides strong evidence that the model with the smaller AIC is considerably better supported by the data [36]. Also, triplet models have shown good promise in distinguishing between competing models [10]. Moreover, we make a causal claim only after comprehensive MR analysis.

A bigger limitation is a lack of statistical power to find more causal relationships, mostly due to our small sample size. Assuming that a genetic instrument (e.g. GRS) describes 5% of the variation of the exposure and a standardized causal effect size between the exposure and the outcome is 0.1, we would need around 15,000 samples to detect an instrument-outcome association with 80% power at nominal significance level 0.05 [56]. We would have to assume a slightly larger causal effect to achieve only 20% power in the Estonian data. It shows that MR is underpowered for hypothesis-free testing in smaller samples. On the other hand, relying on likelihood-based methodology alone can give misleading results due to the number of false positive findings [15, 23]. These can be expensive, time-consuming and difficult to experimentally validate. We think that our approach of combining MR with prior filtering by maximum likelihood modelling can be useful in such cases.

Our analysis strategy could be applied to any trait, but the available sample size in the Estonian Biobank was not sufficient for more complex traits, like BMI and height. A recent MR analysis on summary-level data implicated 68 causal genes for height and 9 for BMI [22], which we attempted to replicate. For height, only 5 genes (out of more than 12,000 tested) cleared the significance threshold at the first analysis step (i.e. significant trait-expression association) in the Estonian data, none of which are among the 68 published causal height genes. Moreover, no gene passed the Δ_AIC_ ≥ 10 threshold filter to indicate a likely causal model. For BMI, only one of the 9 reported causal BMI genes was correlated with BMI in the Estonian data, with ambiguous results in terms of causality. There were again no likely candidates for the causal model. Negative results here can partly be due to different technologies used in quantifying gene expression levels (we used RNA-seq, [22] used microarrays) and partly because BMI and height are far more complex than CRP and require more samples to analyse. Parallel to experimental validation of the CRP-*CD59* link, we also performed summary-level MR in an attempt on an alternative validation of this relationship by using all association summary statistics (*trans* effects based on ~16,000 individuals) between *CD59* expression and CRP-associated SNPs (or their proxies due to data availability) provided by the eQTLGen Consortium. We could not detect a causal effect here, probably again due to low power. Using a single SNP as an instrument and assuming a standardized causal exposure-outcome effect of 0.1, we would need close to one million samples to detect an instrument-outcome association with 80% power.

Our filtering approach is conceptually similar to that of Schadt *et al*. [10] but there is a key difference. Notably, we use gene expression values as the central building blocks to be associated with the phenotype, instead of genotype data. As a result, we have to compute the likelihoods of 4, not 3 possible models, but our approach also has several benefits. First, there are considerably fewer genes than genetic variants, reducing our multiple testing burden. Second, we do not rely on identifying phenotype-associated genetic variants that can be difficult to detect genome-wide due to small effects. The effects of gene expression on the phenotype are likely to be much bigger compared to the effects that individual SNPs have, so in some cases it could be possible to trace the variation in the phenotype back to the SNP only through gene expression [57]. Third, we are able to detect a causal effect from phenotype to gene expression if the colliding model holds. This would not be possible if we required an explicit SNP-phenotype association for the triplets, since under the colliding model that association would not exist. In fact, only our modified approach could identify the CRP-*CD59* link.

In summary, we have demonstrated that combining gene expression data with genotype and phenotype data–and importantly using integrated modelling techniques–can give insight to the causal molecular mechanisms underlying trait variation even if the sample size is limited. Using new RNA-seq data from the Estonian Biobank, we have presented genes that are associated with CRP based on the expression values, identified genetic loci that guide this expression, and provided evidence about the direction of causal effects between CRP and a few genes. Most notably, we have shown by statistical analysis and cell culture stimulation assays that CRP upregulates *CD59* expression in whole blood and can thus have a role in protecting human blood cells from the adverse effects of the immune defence system. We have also presented suggestive evidence of an indirect causal effect of *FADS2* expression to CRP levels. These findings can potentially provide deeper understanding of the functional roles of CRP, but further investigations are required to evaluate these results in terms of chronic inflammation and disease.

## Methods

### Ethics statement

All participants have provided an informed consent for the use of their medical records (www.biobank.ee).

### Estonian Biobank cohort

This study is based on the Estonian Biobank cohort, developed and maintained by the Estonian Genome Center, University of Tartu (EGCUT). This is a volunteer-based population cohort with close to 52,000 participants, which is around 5% of the Estonian adult population. All participants have donated blood samples, 2,700 of which are characterised by clinical biochemistry measurements including the levels of C-reactive protein (CRP, mg/L), leukocytes, erythrocytes and thrombocytes [24]. Recently, whole genomes were sequenced for 2,244 individuals in the EGCUT cohort [25]. Of those, 1,026 have biochemistry measurements and 586 also have RNA-seq data.

### C-reactive protein data

The average CRP value in our data was 2.34 mg/L with standard deviation 3.84. The distribution of CRP measurements was skewed to the right and the maximum CRP value measured was 53.8 mg/L. We have taken a natural logarithm of CRP in this study to bound the effect of slightly outlying values. There was a noteworthy correlation between the levels of CRP and different blood components, most notably white blood cells (p = 2.6×10^−14^), also BMI (p = 6.9×10^−45^) and age (p = 6.5×10^−13^). One individual had missing CRP and blood component values; these were imputed as the corresponding averages of the remaining 1,025 individuals. Four individuals had missing BMI values but all of these originated from follow-up questionnaires so we imputed them from the values given on recruitment a couple of years earlier.

### RNA sequencing data

RNA was extracted from thawed Tempus tubes using TRIzol Reagent (Invitrogen) and further purified using RNeasy Mini Kit (Qiagen). Globin mRNA was depleted using GLOBINclear Kit (Invitrogen). RNA quality was checked using an Agilent 2200 TapeStation (Agilent Technologies). Sequencing libraries were prepared using 200 ng of RNA according to the Illumina TruSeq stranded mRNA protocol. RNA sequencing was performed at the Estonian Genome Center Core Facility using Illumina paired-end 50 bp sequencing technology according to manufacturers specification.

We used Trimmomatic (version 0.36) [58] to remove the adapters and leading and trailing bases with a quality score B. Quality control was done by FastQC (version 0.11.2) [59]. We used STAR (version 2.4.2a) [60] to map the reads to a human genome reference version GRCh37.p13. Concurrently, STAR also counted reads that mapped to each genomic feature using the same algorithm as default htseq-count. In this study, only protein coding genes from autosomal chromosomes were used as evidenced by the Ensembl BioMart (genome assembly GRCh37.p13, release 75) database, the rest were filtered out.

### Data pre-processing

The initial pre-processing and quality control of the WGS data was done by EGCUT as reported in [25]. For our purposes, we performed some further filtering steps using Plink 1.9.0 [61]. We excluded chromosomes X and Y from the analysis and only included those individuals with RNA-seq and CRP data (N = 491). From the remaining sample, genetic variants with minor allele frequency below 0.05 or missing call rates exceeding 0.01 were filtered out. We performed identity by descent analysis (prior to that, we excluded SNPs that were in high pairwise linkage disequilibrium: r^2^ > 0.5 in a sliding window of 50 bases with 5 base increments) which revealed 4 pairs and 1 trio of individuals related to each other (genetic relatedness > 0.1). Only one individual from each group was kept.

As a further quality control measure, we applied MixupMapper [62] to detect and in some cases correct for sample mix-ups. We also performed principal component analysis on the gene expression data and identified a batch of samples with a different gene expression structure compared to other samples. This was discovered to be due to a technical problem during library preparation and affected samples were removed from the analysis.

We also removed non-expressed and lowly expressed genes from the analysis by including only those genes that for at least ten individuals had a count per million (cpm) value greater than 1. After all the filtering steps, the remaining sample size was 491 and the remaining number of genes was 12,619.

### CRP-associated genes

RNA-seq count data is heteroscedastic and that remains the case after the log(cpm) transformation. One of the typical approaches in this case is using weighted linear regression where individual gene expression levels are attributed with weights that are inverse proportional to variance. We thus performed the analysis in the limma framework (version 3.26.9) [63] and found gene expression weights by the voom [26] method that has been shown to work well in differential expression analysis; log(CRP) was used as an exploratory variable and gene expression levels as dependent variables. We adjusted for possible confounding effects from age, BMI, sex and blood components (neutrophils, eosinophils, basophils, lymphocytes, monocytes, erythrocytes and thrombocytes). The first four PCs on the genotype data were used to control for population structure (PCs were again calculated on LD-pruned data) as established in [64]. To account for batch effects in the gene expression data, we used the sequencing batch date as a covariate. Raw RNA-seq counts were normalized with the weighted trimmed mean of M-values [65] method in the edgeR package (version 3.12.1) [66]. Logarithm of count per million was used as the final gene expression measure.

Principal component analysis on the gene expression data revealed hidden batch effects despite controlling for the sequencing batch date. To increase power and the reliability of results, we applied a simple algorithm to account for such hidden effects in a similar fashion to surrogate variable analysis [67] and PEER [68]. We tested whether the top PCs were significantly associated with CRP and decided to use the first two PCs as control variables in the further analysis, because we could see strong associations with CRP starting from the third PC. We adjusted the models for confounders such as age, gender and BMI but also the number of different blood cells to account for differences in gene expression in these cells.

We used Benjamini-Hochberg correction to correct for the number of tests and control the FDR at 0.05. Top genes were subjected to enrichment analysis by g:Profiler [31].

### *Cis*-eQTL analysis

With each of the top genes that were significantly associated with CRP by their expression levels, we performed a *cis*-eQTL analysis. An association between a SNP and a gene was determined only if the SNP resided not farther than 250 kb from the gene. We used the same set of covariates as before, including a batch variable of the WGS data as an additional covariate. The analysis was performed in Plink using ordinary least squares with gene expression measured as log(cpm) as the dependent variable. To control for the number of tests, we used a two-step procedure.

First, we controlled the family wise error rate for each gene by doing 10,000 permutation tests in Plink. However, we did not want to limit our p-values with 1×10^−4^. For each gene, we pulled the highest t-statistic value of every permutation (10,000 in total) and transformed them to p-values. We used the minimum sample size of tested SNPs in the calculation of degrees of freedom, because SNPs contained a variable amount of missing values (but at most 10%) and the SNP that obtained the highest t-statistic was not specified in the Plink output. For each gene, we transformed the 10,000 extreme p-values by −log_10_ and then fitted a Gumbel distribution G(μ, β) on them by estimating μ and β. Finally, nominal p-values p_nom_ were transformed to permutation p-values by p_perm_ = P(X > −log_10_(p_nom_)) where X ~ G(μ, β). This procedure is conceptually very similar to the one implemented in the FastQTL tool, where Beta distribution is used to model the smallest non-transformed p-values [69].

Second, to control for the number of genes tested, we used the Bonferroni method. All SNP and gene expression pairs with permutation p-values less than 0.05/N (N = 1,614 was the number of unique genes tested) were deemed significant.

### Triplet models

We established genes whose expression was associated with CRP and SNPs that were QTL to the expression of those genes. We called these intertwined components triplets. To determine the most likely causal structure underlying these triplets, we performed maximum likelihood modelling in similar fashion to Schadt *et al*. [10], albeit with some differences discussed above. Assuming directed acyclic graphs and the correlation structure within each of the triplets, the following models are possible (Fig 1B):

1. causal: P(G, E, CRP) = P(G)P(E|G)P(CRP|E),
2. colliding: P(G, E, CRP) = P(G)P(CRP)P(E|G, CRP),
3. reactive: P(G, E, CRP) = P(G)P(CRP|G)P(E|CRP),
4. independent: P(G, E, CRP) = P(G)P(E|G)P(CRP|G, E) = P(G)P(CRP|G)P(E|G, CRP).

These models are not Markov equivalent like E -> CRP and CRP -> E in which case the joint distributions would be equal: P(E, CRP) = P(E)P(CRP|E) = P(CRP)P(E|CRP). This means that by calculating the model likelihoods we can determine, for each triplet, the most likely model and hence identify the most plausible causal direction between E and CRP. We found residual CRP from the model log(CRP) = Xb + e and residual expression values from the model log(cpm) = Xb + e where X includes the confounders (age, sex, blood components, PCs). We then used these residuals in the triplet models. We assumed normal distribution for log(cpm) and log(CRP). Wherever necessary, we also assumed multivariate normal distribution and used the appropriate formulas for conditional distributions. We constructed likelihood functions corresponding to each of the above models and maximized them by numerical optimization (optim function in R). Finally, we chose the model with the minimal AIC as the likeliest for each triplet.

### Mendelian randomization analysis

Residual CRP and expression values were used for the analysis of colliding models with MR principles in the Estonian data for consistency of the variables used in the maximum likelihood modelling of triplets. The causal effect between CRP and *CD59* expression was estimated using the tsls function in the R sem package. Summary-level MR analysis was performed using the inverse-variance weighted method [20].

### Cell culture experiments

Human heparinized peripheral blood was diluted with OpTmizer cell culture medium 1:4. The peripheral blood cells from two independent donors were cultivated in three replicates with five increasing CRP (Sigma) doses (50×4^−4^, 50×4^−3^, 50×4^−2^, 12.5 and 50 μg/ml) for 24 and 48 hours. Separate control experiments with 0.1% NaN_3_ in three replicates were included. The cell cultures were stained with phycoerythrin-conjugated anti-human *CD59* antibody (Biolegend) and treated with Lysing solution (BD Biosciences) to eliminate erythrocytes before analysis by flow cytometer (LSRFortessa) and FACSDiva software. Granulocytes were gated according to their forward and side scatter characteristics and *CD59* staining intensity recorded as mean fluorescence index. Approval was obtained from the ethics committee of the University of Tartu.

## Supporting information

S1 Fig Correlations between the expression of all the 1,614 significant CRP-associated genes. A heatmap is shown, depicting correlation strength. Genes are annotated by the sign of their association with CRP and whether they are interferon (IFN) regulated.
(TIF)

S2 Fig Upregulation of *CD59* surface expression by CRP in cell culture experiments after 24 hours. The *CD59* antigen values in mean fluorescent intensity units measured 24 hours after treating peripheral blood cells from two patients with CRP. For negative controls, the cells were not treated with CRP or were treated with additive NaN_3_ only. Black dots represent individual measurements in different replicates, red dots are the averages and whiskers represent ±1 standard errors.
(TIF)

S1 Table All the CRP-associated genes. Association effect sizes with 95% confidence intervals and p-values adjusted for 5% FDR are shown.
(XLSX)

S2 Table Results of the pathway enrichment analysis with g:Profiler. Separate analyses using all the CRP-associated genes, only genes positively correlated with CRP and only genes negatively correlated with CRP.
(XLSX)

S3 Table All the significant *cis*-eQTLs. Association summary statistics from the *cis*-eQTL analysis. The last two columns indicate whether the eQTL was also found in GTEx (V6p) or Westra *et al*. (2013) studies, respectively.
(XLSX)

S4 Table All the triplets with Δ_AIC_ ≥ 2. Summary results from CRP-gene expression association, *cis*-eQTL and triplet model analyses, and p-values from the conditional correlation analysis for triplets with Δ_AIC_ ≥ 10.
(XLSX)

S5 Table g:Profiler results using genes with Δ_AIC_ ≥ 2 in favour of either the causal or colliding models. Each gene’s Δ_AIC_ value was determined based on the triplet with the strongest eQTL.
(XLSX)
