# Supplementary figures and images for: C-reactive protein upregulates the whole blood expression of CD59 - an integrative analysis
Source: PLoS Comput Biol. 2017 Sep 18;13(9):e1005766. doi: 10.1371/journal.pcbi.1005766 (PMC5609773; doi:10.1371/journal.pcbi.1005766)

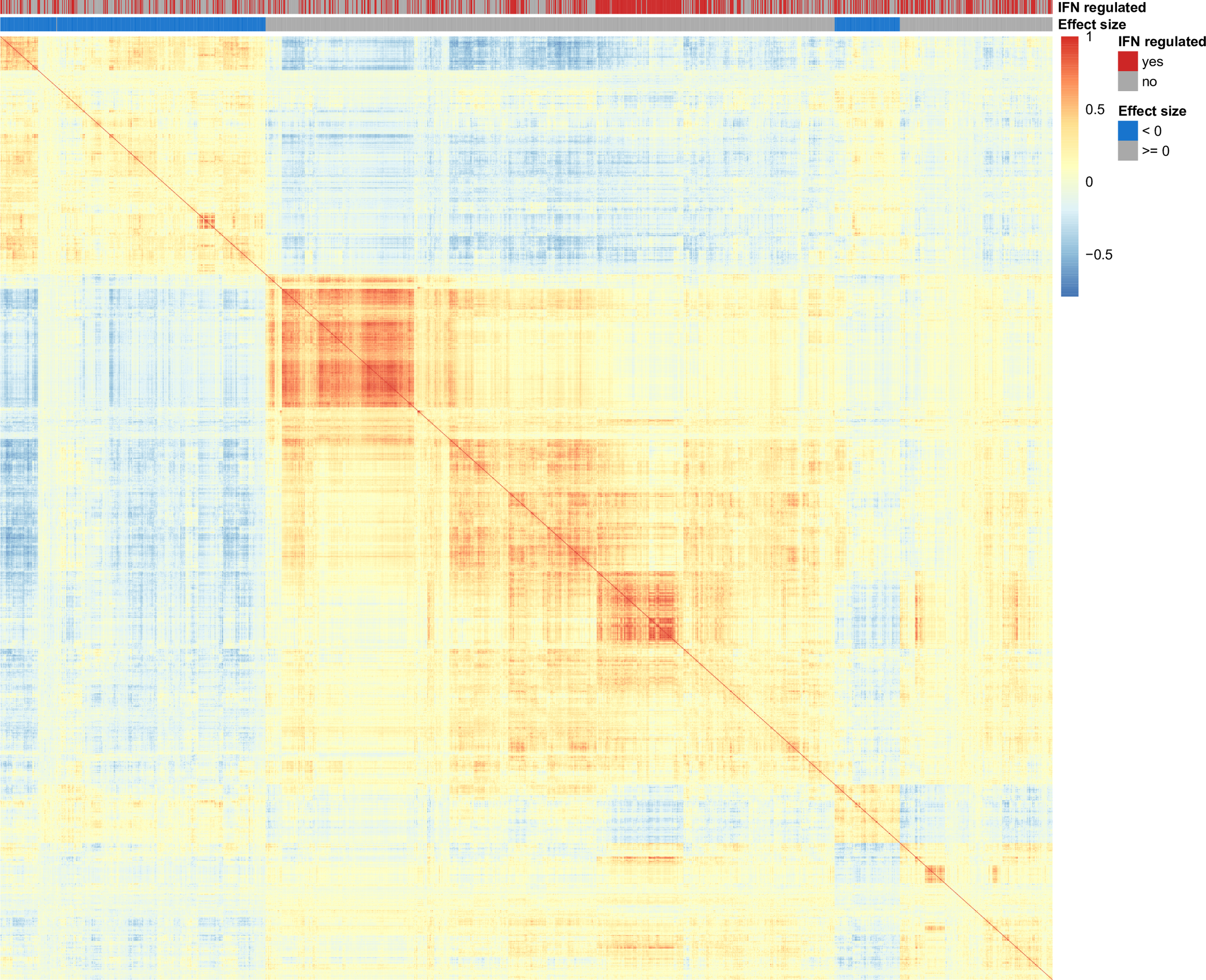

Supplement: S1 Fig — A heatmap is shown, depicting correlation strength. Genes are annotated by the sign of their association with CRP and whether they are interferon (IFN) regulated. (TIF) [file pcbi.1005766.s001.tif]

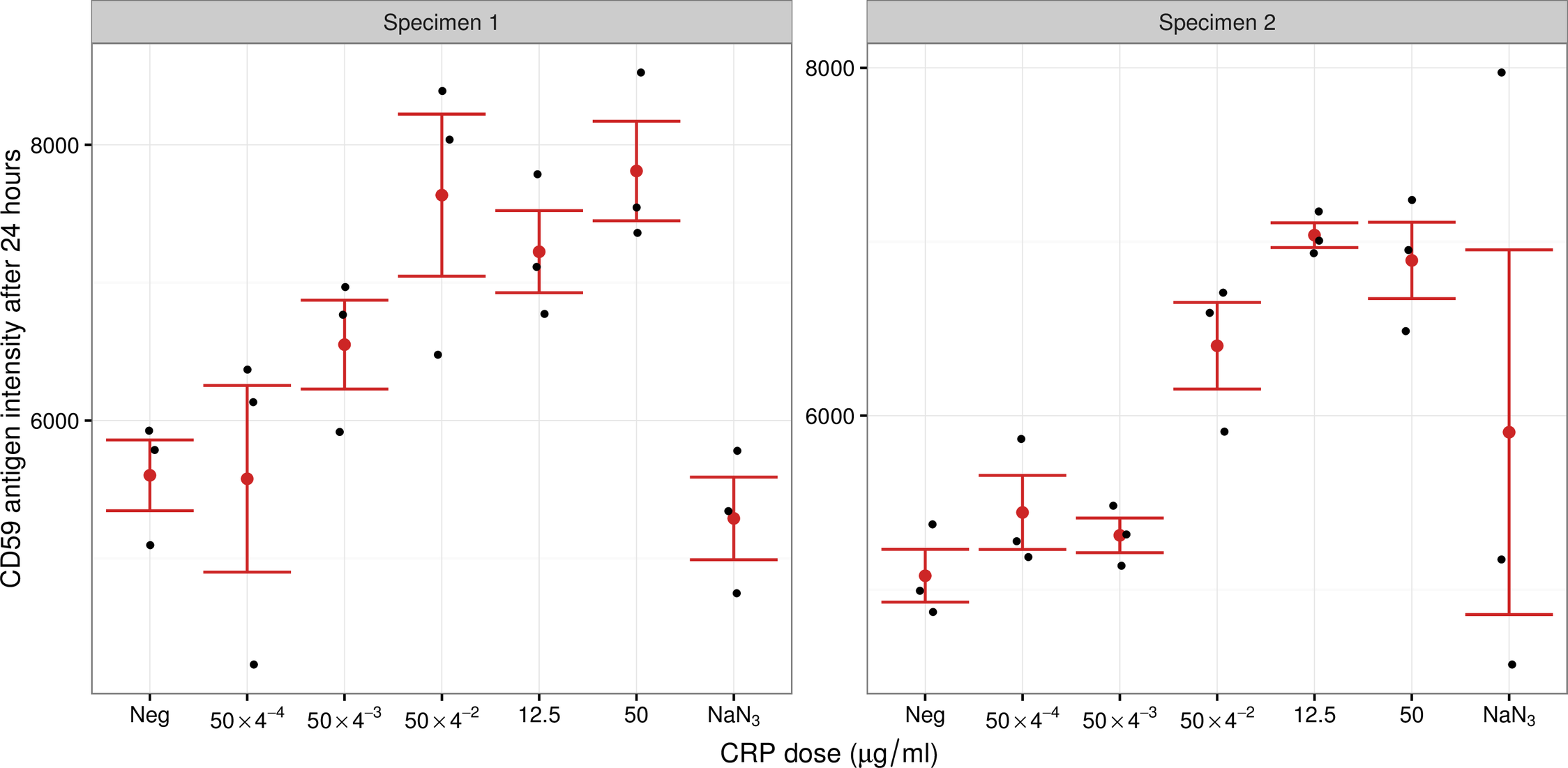

Supplement: S2 Fig — The CD59 antigen values in mean fluorescent intensity units measured 24 hours after treating peripheral blood cells from two patients with CRP. For negative controls, the cells were not treated with CRP or were treated with additive NaN3 only. Black dots represent individual measurements in different replicates, red dots are the averages and whiskers represent ±1 standard errors. (TIF) [file pcbi.1005766.s002.tif]
